# A healthy school start - Parental support to promote healthy dietary habits and physical activity in children: Design and evaluation of a cluster-randomised intervention

**DOI:** 10.1186/1471-2458-11-185

**Published:** 2011-03-25

**Authors:** Gisela Nyberg, Elinor Sundblom, Åsa Norman, Liselotte Schäfer Elinder

**Affiliations:** 1 Division of Intervention and Implementation Research, Department of Public Health Sciences, Karolinska Institutet, Stockholm, Sweden

## Abstract

**Background:**

Childhood obesity is multi-factorial and determined to a large extent by dietary habits, physical activity and sedentary behaviours. Previous research has shown that school-based programmes are effective but that their effectiveness can be improved by including a parental component. At present, there is a lack of effective parental support programmes for improvement of diet and physical activity and prevention of obesity in children.

**Methods/Design:**

This paper describes the rationale and design of a parental support programme to promote healthy dietary habits and physical activity in six-year-old children starting school. The study is performed in close collaboration with the school health care and is designed as a cluster-randomised controlled trial with a mixed methods approach. In total, 14 pre-school classes are included from a municipality in Stockholm county where there is large variation in socio-economic status between the families. The school classes are randomised to intervention (n = 7) and control (n = 7) groups including a total of 242 children. The intervention is based on social cognitive theory and consists of three main components: 1) a health information brochure; 2) two motivational interviewing sessions with the parents; and 3) teacher-led classroom activities with the children. The primary outcomes are physical activity in the children measured objectively by accelerometry, children's dietary and physical activity habits measured with a parent-proxy questionnaire and parents' self-efficacy measured by a questionnaire. Secondary outcomes are height, weight and waist circumference in the children. The duration of the intervention is six months and includes baseline, post intervention and six months follow-up measurements. Linear and logistic regression models will be used to analyse differences between intervention and control groups in the outcome variables. Mediator and moderator analysis will be performed. Participants will be interviewed.

**Discussion:**

The results from this study will show if it is possible to promote a healthy lifestyle and a normal weight development among children from low-income districts with relatively limited efforts involving parents. Hopefully the study will provide new insights to the further development of effective programmes to prevent overweight and obesity in children.

**Trial registration:**

ISRCTN: ISRCTN32750699

## Background

Childhood obesity is multi-factorial and determined to a large extent by dietary habits, physical activity and sedentary behaviours. There is also evidence to suggest that children's physical activity [1], sedentary behaviours [2] and dietary habits [3] to a certain degree track from childhood to adolescence and adulthood, which may lead to health consequences in adulthood [4]. The long-term consequences of childhood obesity include metabolic disturbances, type 2-diabetes and impaired mobility [5]. The more immediate impacts include the child's physical appearance which can result in low self-esteem and lack of self-confidence [5,6]. Due to these serious immediate and future health impacts there is a great interest in obesity prevention programmes in children, which are also more effective than prevention programmes in adults [7]. Most primary prevention trials in children have targeted diet and physical activity habits and have been conducted in pre-schools, schools and homes. A healthy diet and adequate physical activity may be associated with many positive effects of importance for child development such as musculoskeletal, cardiovascular and mental health [8,9].

Recent systematic reviews suggest that school-based interventions to promote diet and physical activity and prevent obesity are more effective if they include a parental component [10,11], but few parental support programmes concerning diet and physical activity habits in children have been evaluated scientifically [12].

It is widely recommended that programme design should be based on theory. Social cognitive theory (SCT) explains behaviour as a reciprocal interaction between personal and environmental factors [13]. Young children have limited abilities to make conscious choices and are therefore strongly dependent on external factors [14-16]. Important factors in the child's physical environment include access to healthy food and opportunities to be physically active. In the social environment, both parents and teachers are crucial in their capacity to encourage healthy eating and physical activity [12]. An important construct in SCT is self-efficacy, which in a parent-child context, may be interpreted as parental self-efficacy to provide support for healthy eating and physical activity. Only a few studies have investigated the associations between parental self-efficacy and diet [14,15], physical activity [16] and sedentary behaviours in children [15].

Motivational interviewing (MI) is a method that can be used within SCT [17]. MI is a client-centred, directive communication style that attempts to enhance a person's intrinsic motivation in order to facilitate behavioural change [18]. There is strong evidence that MI may lead to improved dietary and physical activity habits in adults [19]. We are not aware of any studies on the effectiveness of MI in influencing parents with the aim to influence their own children's lifestyles.

An important aspect to consider in obesity prevention is the family's educational and economic situation. There is a correlation between socio-economic status and the risk of obesity both in adults and in children [20-23]. Studies from Sweden show that children living in deprived areas have about 3-6 times higher risk of becoming obese than children living in affluent areas [24,25]. The overall aim of this study is to develop and evaluate a parental support programme in areas of Stockholm with a high proportion of families with low socio-economic status. Our primary targets are to promote physical activity and healthy dietary habits in six-year-old children starting school. The description of this study protocol follows the CONSORT statement for cluster-randomised trials [26].

## Methods and design

### Study objectives

1) To study the effects of a parental support programme on children's dietary and physical activity habits and weight development.

2) To study mediators and moderators of the effects.

3) To study barriers and facilitators of the intervention.

4) To calculate the costs of the intervention.

### Study design, setting and target group

The "Healthy school start" study is designed as a cluster-randomised controlled trial. The unit of randomisation is school class (pre-school class). The schools are located in a municipality in Stockholm county where there is a large variation in socio-economic status between families. The selected schools are mainly located in low-income areas, but Swedish families are free to select which school their children will attend, independent of home address. There are 29 schools with pre-school classes in the municipality. Twenty-one schools were excluded: six of the schools did not meet the inclusion criteria described below, seven schools declined to participate and eight schools were not included due to the organisation of the school health care in the municipality. Thus, 14 school classes from eight different schools were approached including a total of 320 children. In total, 242 (76%) families agreed to participate in the project.

The study has been approved by the Regional Ethical Review Board in Stockholm (ref: 2010/934-31/1).

### Inclusion criteria

Schools are located in areas with mixed types of housing (blocks of flats, semi-detached houses and detached houses) and with a multi-ethnic population and where about 30% of the parents have no academic education higher than secondary school. In Sweden, all children are offered a place in a pre-school class before they start compulsory school. All families who have a child in a pre-school class in these schools were invited to participate in the project. One of the reasons for choosing a pre-school class is that teachers do not have a strict curriculum to follow and therefore have great flexibility in the classroom. Pedagogical methods from the pre-school and compulsory school are combined in the pre-school class. Further, at this age the children are still dependent on their parents in terms of dietary habits and opportunities for physical activity.

### Exclusion criteria

Schools in affluent areas with a large proportion of families with high socio-economic status are excluded from the study, as are parents/guardians who are unable to communicate in Swedish.

### Planning of the intervention

In the planning stage of the intervention a problem theory and a programme theory were developed according to Fraser et al [27]. Firstly, the problem was specified and SCT was chosen based on previous research and practitioner's experience. In the problem theory we identified parental knowledge, attitude, preference, care and control, role model, willingness to change and parental self-efficacy as malleable factors in order to influence the dietary and physical activity habits and weight development in their children.

**Figure 1:**
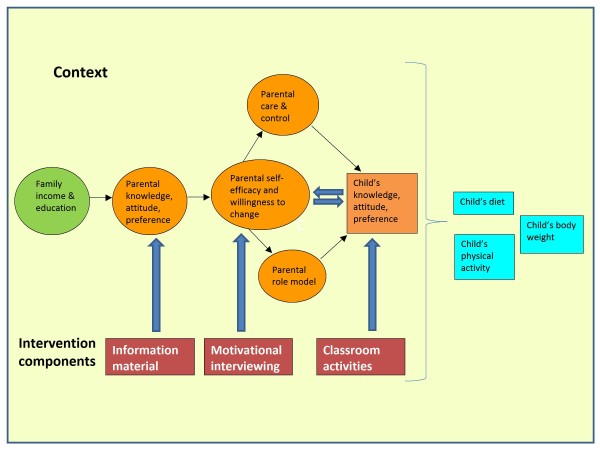
**Conceptual model of parental support intervention**.

A programme theory was developed and the core components of the intervention were identified and combined with the problem theory to a conceptual model of the intervention, see Figure 1.

In the next step, the programme materials were developed and pre-tested. A brochure "A healthy school start - children who are healthy learn well" was developed with the aim of increasing parental knowledge concerning children's dietary, physical activity and sleep habits and how to influence them in a positive direction. The brochure was developed for this intervention and is based on a literature review of evidence-based research regarding parental support for healthy dietary and physical activity habits [28]. We aimed at keeping the text very plain and short with many illustrations. Based on the literature review and the brochure, a teacher's manual and workbooks for the children were developed to facilitate the classroom activities. The materials were developed together with teachers and were inspired by earlier school interventions [29-34]. The children's workbook was constructed so that the "take home" messages would literally be taken home to enforce parents' participation. This workbook has been tested by a group of six-year-old children and their parents.

The organisation of the intervention is as follows. The research team collaborates with two local health promoters and the school physician in the municipality. To begin with, the health promoters contacted the head teachers in the different schools and informed them about the project. Thereafter, the research team visited the teachers in the pre-school class who, together with the head teachers, gave their written consent to participate in the project. Parents were informed verbally about the project at regular school meetings in the spring term or at the beginning of the new school year in the autumn term. The parents were also informed through a letter written by the research team and the school physician. The letter contains easy-to-read information about the intervention and the purpose of the study. The families gave their written consent to participate in the study.

The school classes are randomised after baseline measurements in order to ensure baseline equivalence. After randomisation, the families in both the intervention and control groups will receive a letter informing them about the outcome of the randomisation.

### Intervention components

The duration of the intervention is six months and consists of three main components running in parallel: 1) A brochure is sent home to be read prior to the motivational interviewing session, 2) motivational interviewing with the parents and 3) teacher-led classroom activities with the children.

#### Brochure

The brochure is divided into seven different areas: 1) Parental feeding practices; 2) healthy food and family meal times; 3) physical activity; 4) sweets, snacks, ice-cream and sodas; 5) fruit and vegetables; 6) physical inactivity, screen time, and commercials; 7) sleep. The brochure has been pre-tested by parents and revised according to their suggestions.

#### Motivational interviewing

The parents in the intervention group are offered two sessions of motivational interviewing (MI) during the intervention period. The first session is scheduled to take place in connection with the regular meeting with the school health care staff (nurse and physician) during the child's first school year. This is done partly to minimise the number of times the parents must visit the school and partly to facilitate the integration of the school health care staff into the project. A second session is offered three months later. Each session is planned to last for 45 minutes where the parents discuss issues related to diet, physical activity and sleep with a trained health educator. The health educator uses MI throughout the sessions to enhance the parents' motivation to make changes for their child regarding diet, activity or sleep. A client-centred approach is essential in MI; hence the sessions focus on topics chosen by the parents and proceed in whatever way the parents wish to explore the topics. During the first session the parents are asked to choose, using an agenda-setting tool, an issue regarding their child's diet, activity or sleep that they want to change. As MI is also a directive communication style, the health educator supports the parents by keeping the chosen issue in focus during the session and facilitates the parents' exploration of it using MI techniques. An opportunity to set a goal for the family concerning the matter in focus is given during the first session. During the second session the parents explore their work towards the goal and elaborate on further thoughts regarding the chosen issues. A semi-structured logbook is kept for each session providing information on how the parents perceive the health behaviour of the child, what the parents wish to change or focus on and reasons for wishing the change. The logbook serves as a memory function for the health educator in between the sessions as well as a qualitative source of information about the parents' attitudes and thoughts concerning the health behaviour of their child.

#### Classroom activities

The children are exposed to ten 30-minute teacher-led sessions that convey "take home" messages to enforce parents. The teachers are provided with a tool-box and use the teaching manual for each session. The tool-box contains pedagogic food models made of cardboard, stickers showing the "Green Keyhole" a label for healthy food used in Sweden [35] and illustrated information on how much fruit and vegetables consumption is recommended. It also shows how many discretionary calories children and adults can eat without exceeding energy limits, provided they eat a healthy diet overall. The teachers are also provided with extra educational materials that teach physiology in an easy manner and suggest games that can be used in the classroom or during the breaks. After most sessions the children have homework in their workbooks with the aim to discuss it with their parents/guardians at home. The teachers and children later summarise the homework, so that each theme is repeated with the children.

The control group receives usual teaching in school. Parents and teachers in the control classes will be offered the intervention components after the project is finished.

### Intervention outcomes

Outcomes from the intervention are collected at baseline, after the intervention and at follow-up six months after the intervention.

#### Primary outcomes

Children's physical activity is measured objectively with accelerometers (GT3X, Actigraph LCC, Pensacola, USA) for seven consecutive days. The research team informs the children about the monitor in the classroom and helps them fasten it with an elastic belt on the right side of the hip. The children are instructed to wear the accelerometer for the entire week except while sleeping, bathing or swimming.

Physical activity variables include overall physical activity (average daily counts per minute), average steps per day, time spent in moderate and vigorous intensities and time spent at sedentary intensity averaged per day over the assessment period. The Actigraph accelerometer has been used in many national and international studies to measure physical activity in children and found to be a valid and reliable method [36,37].

Intake of indicator food (fruit/vegetables and energy-dense products), physical activity habits, physical inactivity and sleep will be measured with a parent-proxy questionnaire (Additional file 1). The questionnaire is based on a validated questionnaire from Australia; the Eating and Physical Activity Questionnaire (EPAQ) [38].

Parents' self-efficacy to achieve change is measured by a questionnaire based on a "Tool to measure parenting self-efficacy" (TOPSE) and has been translated and adapted to our research area (diet and physical activity) [39] (Additional file 1). The questionnaire is sent home to parents.

The interviews and focus group discussions will be conducted by an experienced interviewer and moderator without prior contact with the interviewees or focus group participants. A facilitator will be taking field notes during the focus group discussions.

Secondary outcomes are the children's height, weight and waist circumference. Measurements of height and weight are undertaken in school by the research team according to standardised procedures. Height is measured in duplicate to the nearest millimeter using SECA stadiometer (214). Body weight is measured to the nearest 0.1 kilogram using a digital scale (SECA Robusta 813). Waist circumference (WC) is measured in duplicate midway between the lower rib and the iliac crest at the end of gentle expiration. Body mass index (BMI) is calculated as weight (kg) divided by height (m) squared. Overweight and obesity are determined according to the International Obesity Task Force age and sex-specific cut-off points [40] and underweight according to the work of Cole et al [41] derived from the same dataset. BMI standard deviation score is calculated according to Karlberg et al [42].

### Fidelity criteria

Fidelity in this study is assessed with regard to the intervention components. The compliance with the teaching sessions and workbook completion is monitored by questions and regular contact with the teachers. The teachers have a check list where they document how much time they have spent on each session. The parents are asked at the MI session if they have read the brochure.

The level of MI used in the sessions is assessed through expert rating at the Motivational Interviewing Coding Laboratory at Karolinska Institutet. Audio files of sessions are sent to the laboratory on a regular basis. The files are coded and rated using Motivational Interviewing Treatment Integrity (MITI) Code instrument, a behavioural coding system that assesses how well a practitioner performs MI in a session [43].

#### Barriers and facilitators

Experiences from the classroom activity will be collected by discussions with parents and teachers in four focus groups (4-8 in each group) after the intervention period. The aim of the focus group discussion is to elicit the parents' and the teachers' experiences and views on the project, find out if the intervention components have been satisfactory, identify possible barriers and determine whether there is a need for additional components in the intervention, e.g. group activities. Parents' experiences and thoughts about MI will be evaluated through individual semi-structured interviews.

### Statistical power

The power calculation for this study was based on the assumption of an average 20% increase of physical activity assessed as steps per day by accelerometry. Physical activity data (steps per day) from Swedish children were used for the power calculation [44]. The estimated sample size was calculated for a two-sided test with the significance level of 0.05 and power was set to 90% using "sample size calculator for cluster randomised trials" [45]. The calculation shows that 12 school classes with a participation rate of 60% in each class, approximately 144 children in total, are needed to detect a significant change in physical activity between the intervention and control groups. It was decided to randomise 7+7 classes with approximately 200 families in total (100 families in the intervention group and 100 families in the control group) to have a margin for drop-outs and withdrawals. The physical activity variable "time spent sedentary" requires fewer school classes.

### Data analysis

Data analysis will be performed by a statistician who is blinded to the assignment of the intervention and control groups. The study will be evaluated with both quantitative and qualitative methods. Data from the study will be analysed with parametric and non-parametric tests depending on the distribution of the quantitative variables. The analysis will be carried out using the observed cases population and the full analysis population. Linear and logistic regression models will be used to analyse differences between intervention and control groups in the outcome variables physical activity, intake of indicator foods and body weight status with correction for the cluster variance (school class as random factor). Mediators (parental self-efficacy) and moderators (socio-economic status and ethnicity) will be analysed according to MacKinnon [46].

The interviews and focus group discussions will be recorded and transcribed [47,48]. Content analysis will be applied to the transcriptions and field notes. These will be analysed by two members of the research team.

Costs will be calculated by an economist and will be based on how much it costs the school and the municipality to run the project as performed excluding the cost of research.

## Discussion

The results from this innovative programme will show if it is possible to promote a healthy lifestyle and a normal weight development among children from areas with low socio-economic status with relatively limited efforts aimed at parents. Previous research has shown that school-based programmes are effective in promoting healthy dietary habits and physical activity and that effectiveness can be improved by including a parental component [10,11]. School health care in Sweden is in great need of effective programmes and practices to be able to communicate with children and parents about overweight and obesity [49]. A recent study from Sweden shows that a majority of the parents stated a need for support to be able to give their 6-year-old children healthy meals and enough physical activity [50].

There is a need of programmes targeting groups with low socio-economic status. This cluster-randomised trial is novel in investigating whether a combination of motivational interviewing directed at parents and a classroom component directed at children can promote healthy dietary habits and physical activity in six-year-old children living in mainly low-income areas in a municipality in Stockholm county. Interviews with the participants will provide valuable information guiding further development of effective prevention programmes against overweight and obesity in children. The cost calculation will be useful for municipalities planning to implement the programme in the school setting, though further analysis of health impacts will be required to assess its overall cost-effectiveness. If combined with interventions directed at the school environment, this programme may lead to improved health and positive health effects in the short and long term. Final results from this intervention study are expected in late 2012.

## Competing interests

The authors declare that they have no competing interests.

## Authors' contributions

GN, LSE and ES conceived the study and applied for funding, designed the intervention and the evaluation. GN, ES and ÅN performed the data collection. ÅN conducted the MI. GN prepared the initial draft of the manuscript and the other authors have contributed. GN in collaboration with a statistician designed the statistical analysis. All the authors have critically reviewed and approved the final version of the manuscript. All the authors declare that they have no conflict of interest.

## Pre-publication history

The pre-publication history for this paper can be accessed here:

http://www.biomedcentral.com/1471-2458/11/185/prepub

## Supplementary Material

Additional file 1 **A questionnaire for parents' self-efficacy and their children's intake of indicator food, physical activity habits, physical inactivity and sleep**. Click here for file
