# Supplementary material for: A healthy school start - Parental support to promote healthy dietary habits and physical activity in children: Design and evaluation of a cluster-randomised intervention
Source: BMC Public Health. 2011 Mar 25;11:185. doi: 10.1186/1471-2458-11-185 (PMC3071321; doi:10.1186/1471-2458-11-185)
Supplement: Additional file 1 — A questionnaire for parents' self-efficacy and their children's intake of indicator food, physical activity habits, physical inactivity and sleep. [file 1471-2458-11-185-S1.DOC]

First page of the original Swedish questionnaire which is translated below

**********

**Questions about your child´s dietary and physical activity habits**

Please answer the questionnaire on a Tuesday, Wednesday or a Thursday. Think about habits in the home. If the child lives alternately with Mom and Dad we ask you each to fill in a separate survey.

1. Name of the child …………………………………………
   1. Girl
   2. Boy
2. Personal identification number ………………………
3. Who is filling in the questionnaire? Mother Father
4. How many people live in the same household as the child? (If the child lives in two different households, please answer both question number 4 and 6) ………………
5. Type of housing
   1. Rented flat
   2. Owned flat
   3. House
   4. Semi-detached house
   5. Other, please specify?..................

If the child lives alternately with Mom and Dad:

1. How many people live in the other household?
2. Type of housing
   1. Rented flat
   2. Owned flat
   3. House
   4. Semi-detached house
   5. Other, please specify?..................

8. What is the highest education level of the child´s **mother**?

1. High school or elementary school
2. 2-year college or vocational school
3. 3-4 year college
4. University
5. Other education? ………………………

9. What is the highest education level of the child´s **father**?

1. High school or elementary school
2. 2-year college or vocational school
3. 3-4 year college
4. University
5. Other education? ………………………

10. The child´s **mother** was born in:

1. Sweden or Scandinavia
2. Europe
3. Outside Europe

11. The child´s **father** was born in:

1. Sweden or Scandinavia
2. Europe
3. Outside Europe

12. **Yesterday**, how long time did your child watch TV/videos/DVD or play computer – or video-games at home? (or at a friend´s or relative´s home)?

Morning ………hrs ………minutes Don’t know

Afternoon ………hrs ………minutes Don’t know

Evening ………….. hrs ………minutes Don’t know

13. **Yesterday**, how many servings of the following beverages did your child drink? (1 serving = 1,5 dl)

Fruit juice *None 1 2 3 4 5 6 or more Don´t know*

Cordial or soft drink *None 1 2 3 4 5 6 or more Don´t know*

Water *None 1 2 3 4 5 6 or more Don´t know*

Plain milk *None 1 2 3 4 5 6 or more Don´t know*

(include milk on cereal or porridge)

Flavoured milk, hot chocolate *None 1 2 3 4 5 6 or more Don´t know*

14. **Yesterday**, how many servings of the following foods did your child have?

Vegetables (1 serving = 2 dl grated carrots/cabbage or a big tomato or 2-3 broccoli stalks)

*None 1 2 3 4 5 6 or more Don´t know*

Chips / cheese doodles (1 serving = 1,5 dl)

*None 1 2 3 4 5 6 or more Don´t know*

Fruit (a small apple or a bunch of grapes (about 10) )

*None 1 2 3 4 5 6 or more Don´t know*

Confectionary, chocolate (about 1,5 dl candy or 4 pieces from a of chocolate bar)

*None 1 2 3 4 5 6 or more Don´t know*

Sweet biscuits, cakes, buns (1 serving = a small bun, or 5 small biscuits)

*None 1 2 3 4 5 6 or more Don´t know*

Ice cream (1 serving = a small popsicle stick or 1 dl ice cream)

*None 1 2 3 4 5 6 or more Don´t know*

15. How many servings of vegetables does your child usually eat each day? (1 serving = 2 dl grated carrots/cabbage or a big tomato or 2-3 broccoli stalks)……………………

16. How often does your child eat takeaway or fast-food? (eg hamburgers, kebab pizza , tacos)

1. Less than once a month
2. 1-3 times per month
3. Once a week
4. 2-4 times per week
5. 5-6 times per week
6. Once a day
7. 2 or more times per day

17. What kind of milk does your child drink at home?

1. Whole milk (3 % fat)
2. Low-fat milk (1.5 % fat)
3. Skimmed milk (0.5 % fat)
4. Other kind, please specify…………………………
5. Don´t know

18. Last week, how many times did you or a family member take your child to a playground, park, swimming pool, or other place for physical activity?

……….times last week

19. What does your child usually do when she/he has a choice about how to spend free time?

1. Usually chooses inactive pastimes (eg TV, computer, drawing or reading)
2. Just as likely to choose inactive as active pastimes
3. Usually chooses active pastimes (eg outdoor play, dancing, sports)

20. Is your child a member of a sports club?

Yes No Don´t know

What sport?.........................................

Times per week? ……………………………………….

21. How many hours does your child usually sleep?

1. 6-7 hours
2. 7-8 hours
3. 8-9 hours
4. 9-10 hours
5. More than 10 hours
6. Don’t know

Finally some questions about your role as a parent and how you perceive your ability to handle different situations with your child.

***Mark the alternative that most accurately describes the situation.***

***For the child´s eating habits***

1 2 3 4 5 6 7 8 9 10

Not at all Very little Some influence Quite a bit A great deal Very much

*(In the original Swedish version, a Likert scale ranging from ”Not at all” to “Very much” or “Never” to “Always” is repeated for every statement below)*

22. I can give my child good eating habits

23. I can have enjoyable mealtimes with my child

24. I can arrange regular meals at home

25. I can arrange that we eat meals together

26. I can offer my child meals that contain fruit and vegetables

27. I can limit the number of occasions we eat at fast-food restaurants to every other week

28. I can remain calm when facing difficulties at mealtimes

29. I can establish limits for my child´s consumption of sweets, ice cream, biscuits, crisps etc

30. I am able to stick to the rules I set up for my child

***For the child´s physical activity***

31. I can see to it that my child is physically active

32. I am able to have fun with my child outdoors

33. I am able to arrange outdoor activities for my child (eg outdoor games, bicycling, sports activities)

34. I can plan activities that my child will enjoy

35. I understand my child´s needs when it comes to sports and physical activities

36. I can set limits for how much my child watches TV

37. I can influence when my child goes to bed and how much my child sleeps

38. I am able to stick to the rules I set up for my child

**********

Thank you!

Any questions, please contact Elinor Sundblom

[Elinor.sundblom@ki.se](mailto:Elinor.sundblom@ki.se)

08-524 82668
